# Supplementary material for: Molecular Characterization of White Wines Antioxidant Metabolome by Ultra High Performance Liquid Chromatography High-Resolution Mass Spectrometry
Source: Antioxidants (Basel). 2020 Jan 28;9(2):115. doi: 10.3390/antiox9020115 (PMC7070782; doi:10.3390/antiox9020115)
Supplement: Supplementary file 1 [file antioxidants-09-00115-s001.pdf]

Molecular characterization of white wines antioxidant metabolome by ultra high performance liquid chromatography high-resolution mass spectrometry

Remy Romanet, Florian Bahut, Maria Nikolantonaki* and Régis D. Gougeon

Université de Bourgogne Franche-Comté, AgroSup Dijon, PAM UMR A 02.102, Institut Universitaire de la Vigne et du Vin, Jules Guyot, Rue Claude Ladrey, BP 27877, 21078 Dijon Cedex, France

* Corresponding: maria.nikolantonaki@u-bourgogne.fr

Supplementary Data

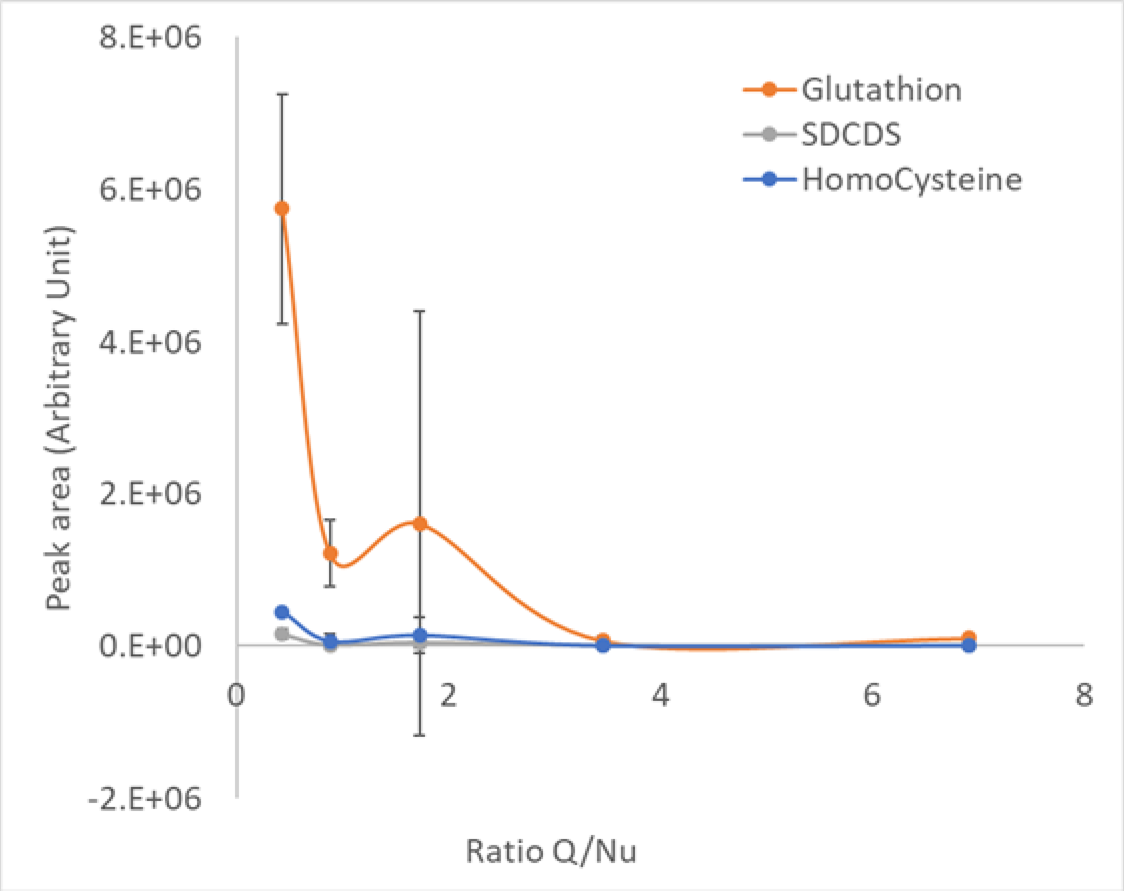

Figure 1. Peak area of tested sulfur compounds in native form for different quantity of 4MeQ, explain as ratio of quinone and total nucleophilic compounds in mol. Met, Glu and Asp-Met were not plot here, because the corresponding derivatized compounds was not detected. No trace of Cys in native from has been detected. .

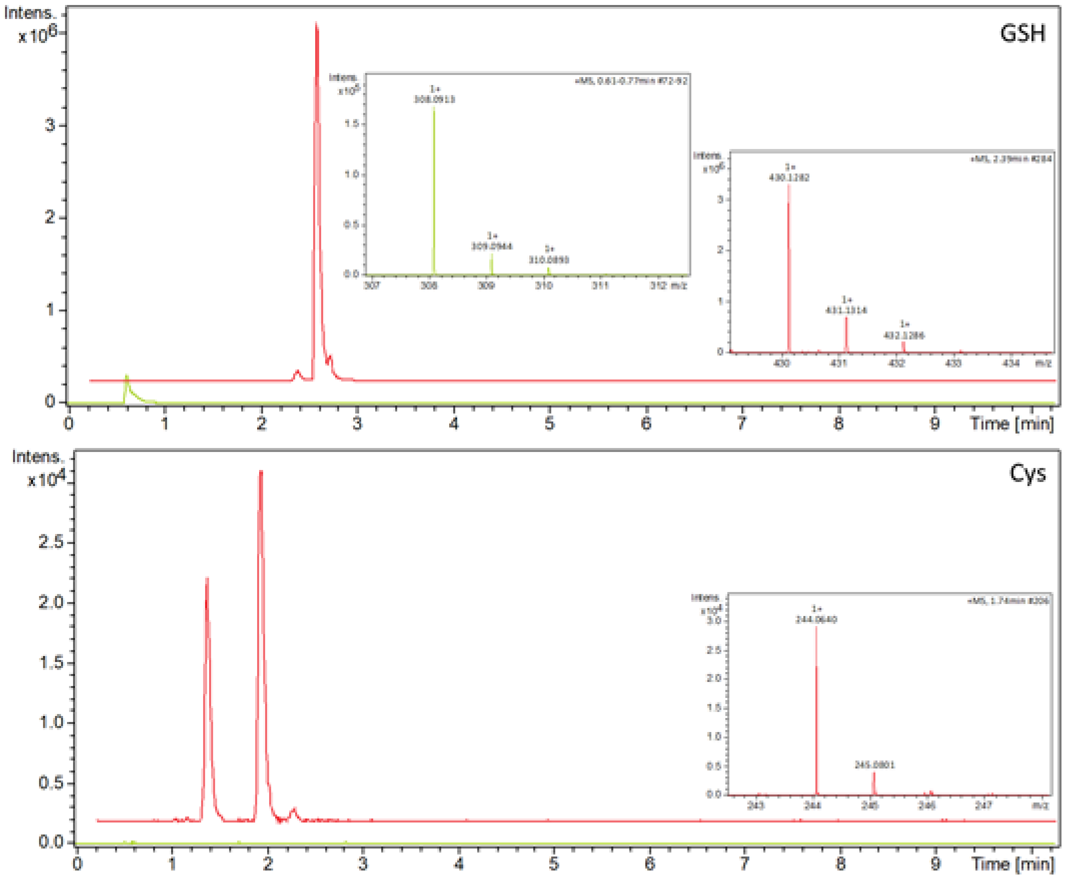
GSH
Intens.
x10^6
Time [min]
308.0913
309.0944
310.0893
430.1282
431.1314
432.1286
Cys
Intens.
x10^4
Time [min]
244.0640
245.0801

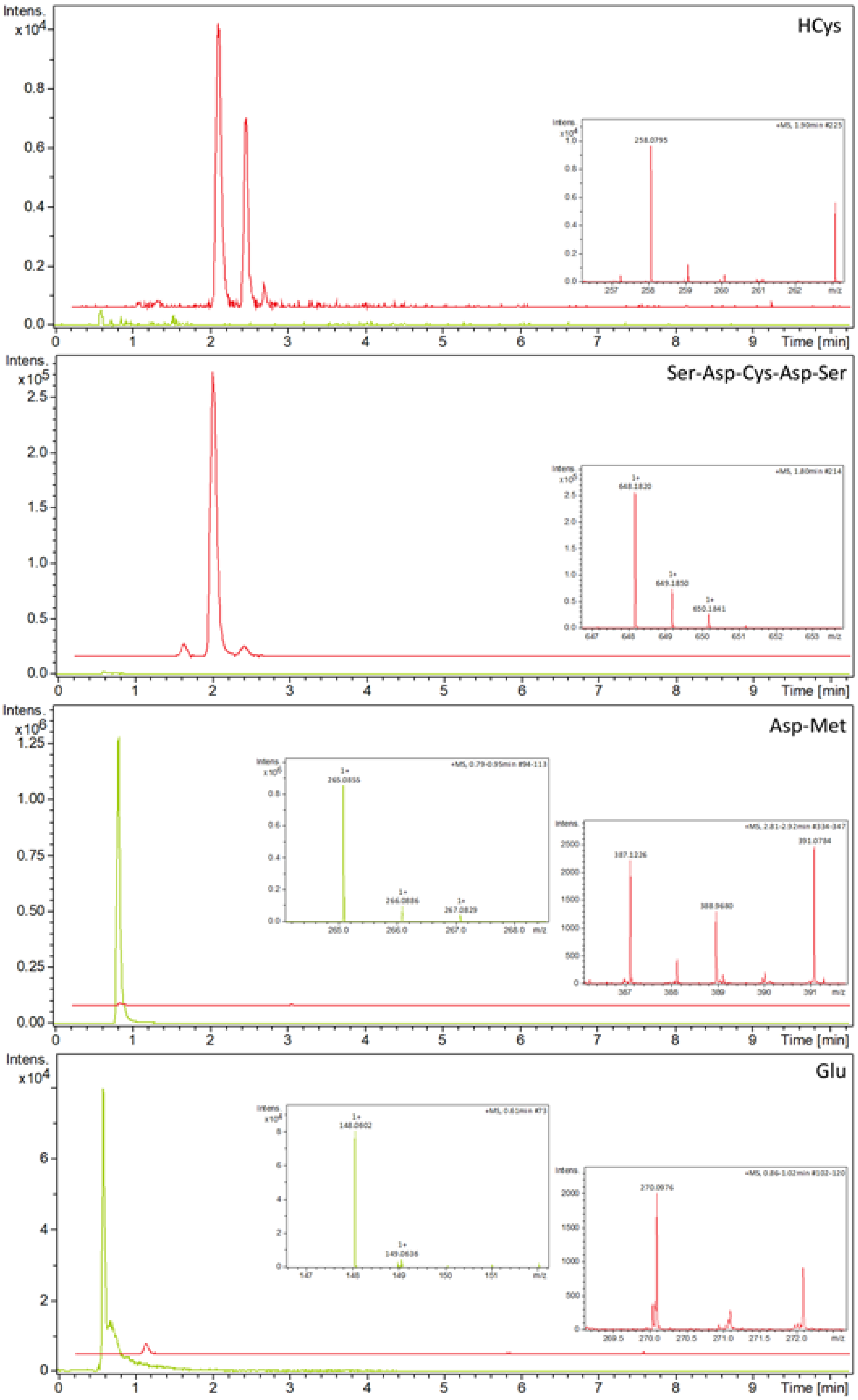

HCys
Intens. x10^4
Time [min]
258.0795
Ser-Asp-Cys-Asp-Ser
Intens. x10^5
Time [min]
648.1820
649.1850
650.1841
Asp-Met
Intens. x10^6
Time [min]
265.0855
266.0886
267.0829
387.1226
388.9680
391.0784
Glu
Intens. x10^4
Time [min]
148.0602
149.0636
270.0976

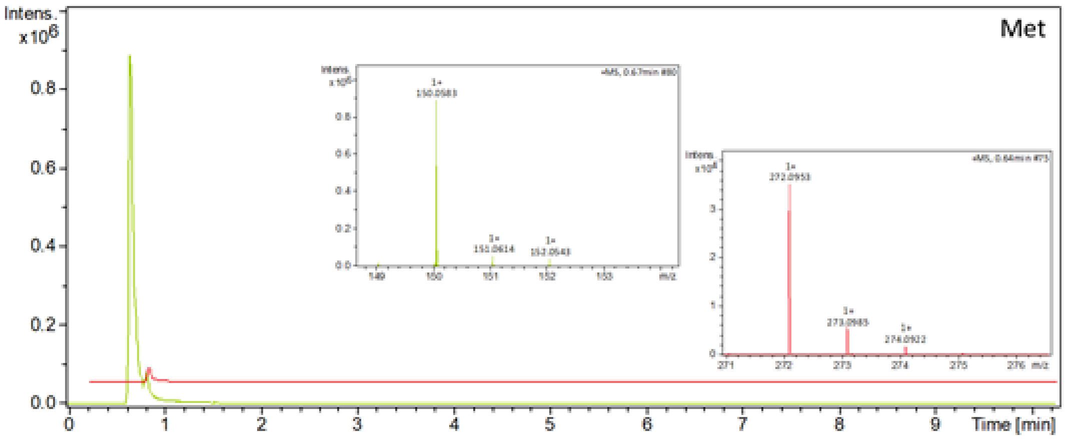

Figure S1 Visualization of the extract ions chromatograms and mass spectrum obtained for standard compounds (GSH, Cys, HCys and Ser-Aps-Cys-Asp-Ser, Asp-Met, Met and Glu) **without (green line) and with derivatizatio**n (red line) using 4MeQ.

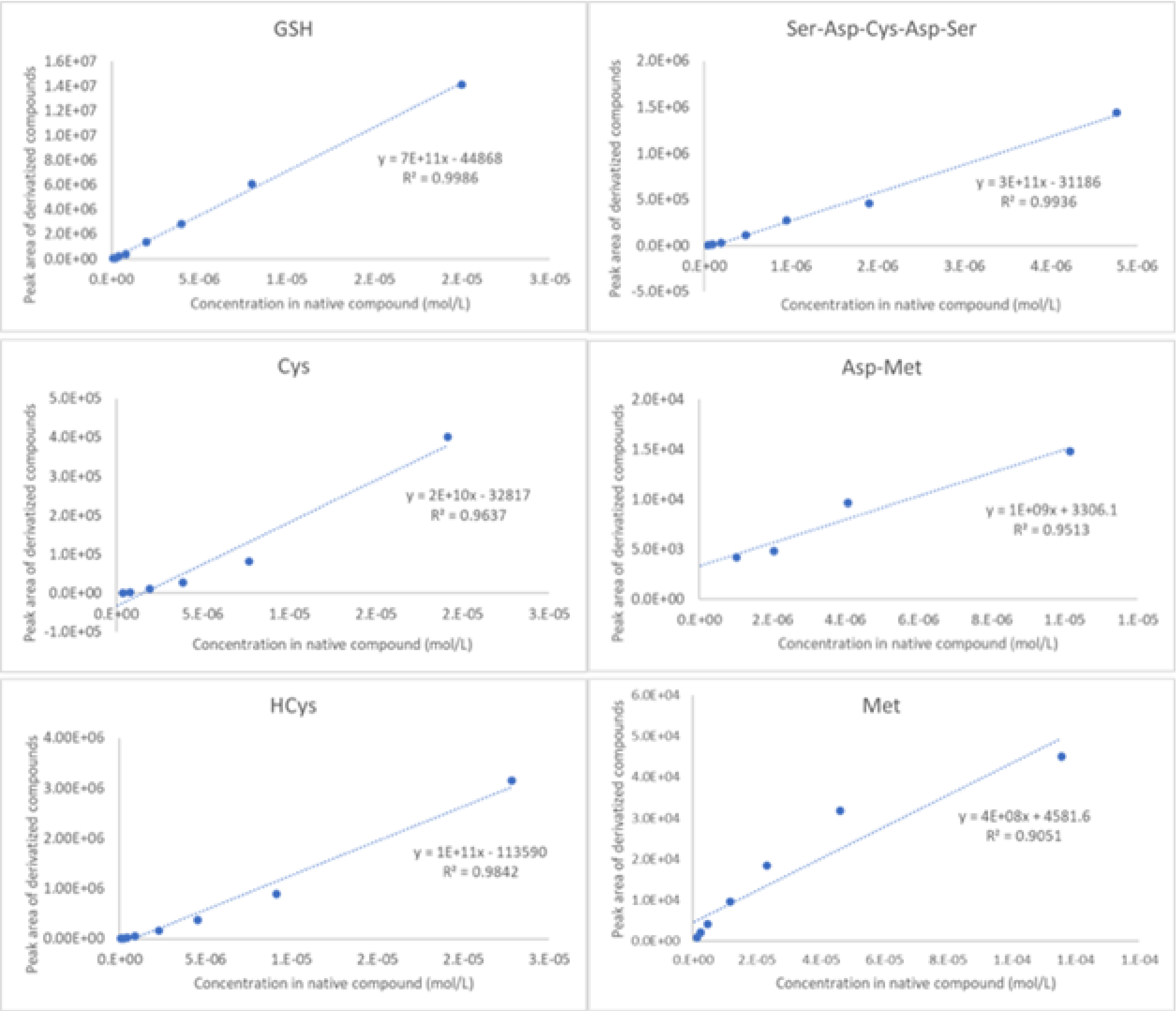

Figure 3. Representation of the area of derivatized compounds against the concentration of the native compounds (**GSH, Cys, HCys and Ser-Aps-Cys-Asp-Ser, Asp-Met and Met)**. Glu have not been plotted because, it was not detected under our experimental conditions (pH 3).

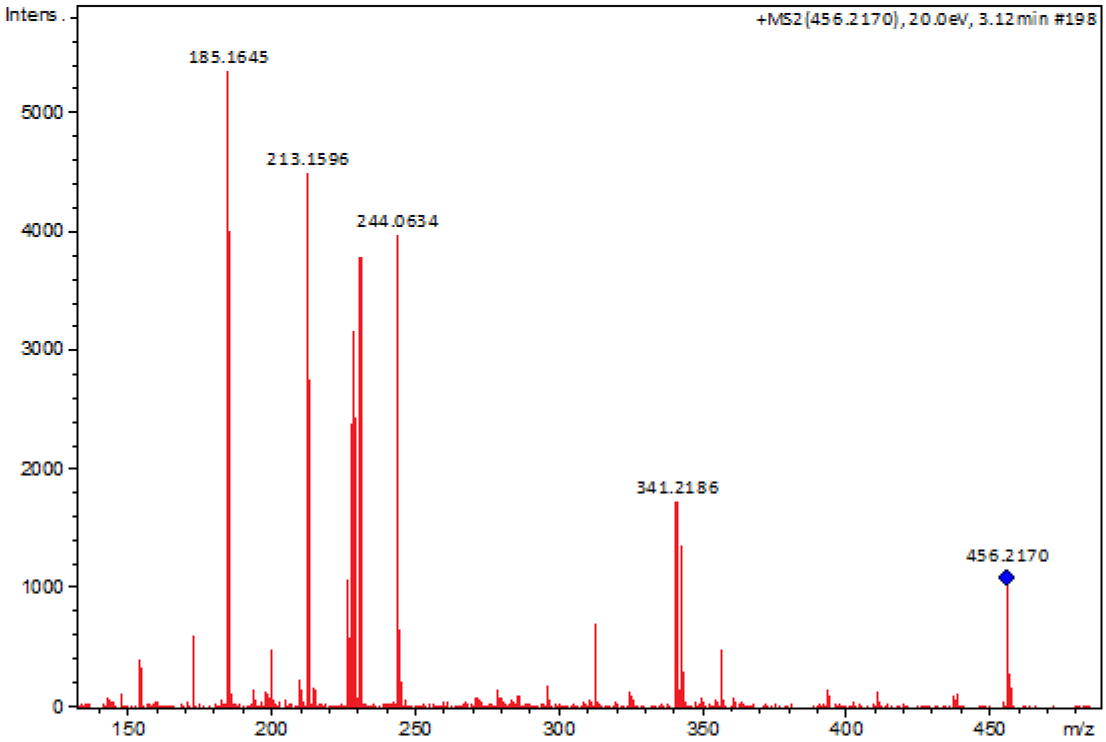

Figure 4. Mass spectra obtain by fragmentation of unknown adduct $C_{14}H_{27}N_3O_4S + 4MeQ$ (456.2156 m/z) at 20 eV. The blue square indicates the molecular ion.

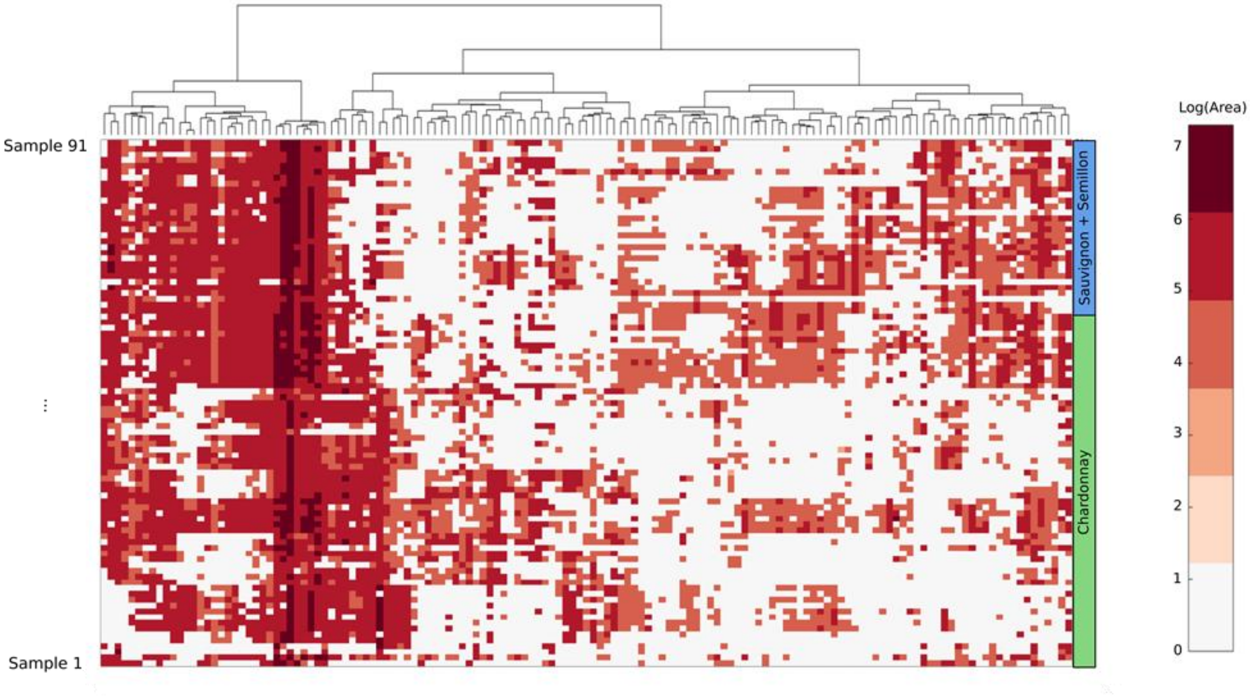

Figure 5. Representative heatmap visualizing the intensities of the 141 derivatized compounds detected in the 91 white wines analyzed (Table S1). Missing values are shown in gray.

antioxidants

MDPI

Table 1. Overview of global wine sample set.

Sample	Winery	Grape variety	Vintage	Aging [a]
1	CN	Chardonnay	2017	After AF
2	CN	Chardonnay	2017	After AF
3	CN	Chardonnay	2017	After AF
4	CN	Chardonnay	2017	After AF
5	Lf	Chardonnay	2017	After AF
6	Lf	Chardonnay	2017	After AF
7	Lf	Chardonnay	2017	After AF
8	Lf	Chardonnay	2017	After AF
9	Lf	Chardonnay	2017	After AF
10	Lf	Chardonnay	2017	After AF
11	Lf	Chardonnay	2017	After AF
12	Lf	Chardonnay	2017	After AF
13	Lf	Chardonnay	2017	After AF
14	Lf	Chardonnay	2017	After AF
15	P	Chardonnay	2016	1 years in barrel
16	P	Chardonnay	2016	1 years in barrel
17	P	Chardonnay	2016	1 years in barrel
18	P	Chardonnay	2016	1 years in barrel
19	P	Chardonnay	2016	1 years in barrel
20	P	Chardonnay	2016	1 years in barrel
21	P	Chardonnay	2016	1 years in barrel
22	P	Chardonnay	2016	1 years in barrel
23	P	Chardonnay	2017	After AF
24	P	Chardonnay	2017	After AF
25	P	Chardonnay	2017	After AF
26	P	Chardonnay	2017	After AF
27	P	Chardonnay	2017	After AF
28	P	Chardonnay	2017	After AF
29	P	Chardonnay	2017	After AF
30	Lf	Chardonnay	2016	1 years in barrel
31	Lf	Chardonnay	2016	1 years in barrel
32	Lf	Chardonnay	2016	1 years in barrel
33	Lf	Chardonnay	2016	1 years in barrel
34	Lf	Chardonnay	2016	1 years in barrel
35	Lf	Chardonnay	2016	1 years in barrel
36	B	Chardonnay	2017	After AF
37	B	Chardonnay	2017	After AF
38	B	Chardonnay	2017	After AF
39	B	Chardonnay	2017	After AF
40	B	Chardonnay	2017	After AF
41	B	Chardonnay	2017	After AF
42	B	Chardonnay	2017	After AF
43	B	Chardonnay	2017	After AF
44	B	Chardonnay	2017	After AF
45	B	Chardonnay	2017	After AF
46	B	Chardonnay	2017	After AF
47	B	Chardonnay	2017	After AF
48	Lf	Chardonnay	2016	After AF
49	P	Chardonnay	2016	After AF
50	Lf	Chardonnay	2017	After AF
51	Lf	Chardonnay	2017	After AF
52	Lf	Chardonnay	2017	After AF
53	Lf	Chardonnay	2017	After AF
54	Lf	Chardonnay	2017	After AF
55	Lf	Chardonnay	2017	After AF
56	Lf	Chardonnay	2017	After AF
57	P	Chardonnay	2017	After AF
58	P	Chardonnay	2017	After AF
59	P	Chardonnay	2017	After AF
60	P	Chardonnay	2017	After AF

61	P	Chardonnay	2017	After AF
62	P	Chardonnay	2017	After AF
63	Ltm	Sémillon	2017	After AF
64	Ltm	Sémillon	2017	After AF
65	Ltm	Sémillon	2017	After AF
66	Ltm	Sémillon	2017	After AF
67	Y	Sauvignon	2017	After AF
68	Y	Sauvignon	2017	After AF
69	Y	Sauvignon	2017	After AF
70	Y	Sauvignon	2017	After AF
71	Y	Sauvignon	2017	After AF
72	Y	Sauvignon	2017	After AF
73	Y	Sauvignon	2017	After AF
74	Y	Sauvignon	2017	After AF
75	Shl	Sauvignon	2017	After AF
76	Shl	Sauvignon	2017	After AF
77	Shl	Sauvignon	2017	After AF
78	Shl	Sauvignon	2017	After AF
79	Shl	Sauvignon	2017	After AF
80	Shl	Sauvignon	2017	After AF
81	Shl	Sauvignon	2017	After AF
82	Shl	Sauvignon	2017	After AF
83	Shl	Sauvignon	2017	After AF
84	Shl	Sauvignon	2017	After AF
85	L	Sémillon	2017	After AF
86	L	Sémillon	2017	After AF
87	M	Sauvignon	2017	After AF
88	M	Sauvignon	2017	After AF
89	M	Sauvignon	2017	After AF
90	M	Sauvignon	2017	After AF
91	M	Sauvignon	2017	After AF
92	M	Sauvignon	2017	After AF

[a] After AF: analysis just after alcoholic fermentation.
